# Sequencing and Characterization of Mitochondrial Protein-Coding Genes for *Schizothorax niger* (Cypriniformes: Cyprinidae) with Phylogenetic Consideration

**DOI:** 10.1155/2020/5980135

**Published:** 2020-12-07

**Authors:** Tasleem Akhtar, Ghazanfar Ali, Nuzhat Shafi, Wasim Akhtar, Abdul Hameed Khan, Zahid Latif, Abdul Wali, Syeda Ain-ul-Batool, Abdul Rehman Khan, Sadia Mumtaz, Syed Iftikhar Altaf, Sundus Khawaja, Sadia, Madiha Khalid, Fazal Ur Rehman, Qudir Javid

**Affiliations:** ^1^Department of Biotechnology, University of Azad Jammu and Kashmir, Muzaffarabad, Pakistan; ^2^Department of Zoology, University of Azad Jammu and Kashmir, Muzaffarabad, Pakistan; ^3^Department of Botany, University of Azad Jammu and Kashmir, Muzaffarabad, Pakistan; ^4^Faculty of Life Sciences & Informatics, BUITEMS, 87100 Quetta, Pakistan; ^5^Department of Chemistry, University of Azad Jammu and Kashmir, Muzaffarabad, Pakistan; ^6^Department of Biotechnology, Women University Bagh, Pakistan; ^7^Department of Microbiology, University of Balochistan, Quetta 87300, Pakistan

## Abstract

The present study was conducted to get more information about the genome and locate the taxonomic position of *Schizothorax niger* in Schizothoracinae through mitochondrial 13 protein-coding genes (PCGs). These PCGs for *S. niger* were found to be 11409 bps in length ranging from 165 (ATPase 8) to 1824 bps (NADH dehydrogenase subunit 5) and encode 3801 amino acids. In these PCGs, 4 genes overlap on the similar strands, while one shown on the opposite one: ATPase 6+8 and NADH dehydrogenase subunit 4+4L overlap by 7 nucleotides. Similarly, ND5-ND6 overlap by 4 nucleotides, while ATP6 and COIII overlap by 1 nucleotide. Similarly, four commonly used amino acids in *S. niger* were Leu (15.6 %), Ile (10.12 %), Thr (8.12 %), and Ala (8.7 %). The results presented that COII, COIII, NDI, ND4L, and Cytb had substantial amino acid conservation as compared to the COI gene. Through phylogenetic analysis, it was observed that *S. niger* is closely linked with *S. progastus*, *S. labiatus*, *S. plagiostomus*, and *S. nepalensis* with high bootstrap values. The present study provided more genomic data to know the diversity of the mitochondrial genome and its molecular evolution in Schizothoracinae.

## 1. Introduction

Nowadays, mtDNA has been frequently used for species identification; it has phylogenetic, evolutionary, and population studies [1–3]. The vertebrate mtDNA is 16–20 kb in size and consists of 37 genes that coded 13 PCGs, 22 transfer RNAs, 2 ribosomal RNAs, and a d-loop region to check its replication and transcription [4, 5]. Mitochondrial genome and its gene contents in fish are quite conserved with few exceptions in the rearrangement of genes [6, 7]. The genome or gene-based studies are helpful for the better understanding of evolutionary and phylogenetic relationships among fishes [3, 8].

Recently, molecular and phylogenetic studies are helpful to solve some phylogenetic questions and persistent discrepancies among teleosts, for example, in the Cyprinidae [9]. Similarly, the evolutionary background of higher teleosts has also been explored through mitogenome studies [10]. For the analysis of phylogenetic trees, the information obtained through a single gene is mostly insufficient [11]. In the cyprinids, the phylogenetic approaches are based on the whole mitochondrial genome or the functional genes (13 PCGs) that are helpful for a better understanding of speciation and divergence [12]. Schizothoracinae (Cyprinidae) fishes represent the largest and most diverse taxon possessing more than 100 species and subspecies (http://www.fishbase.org) with a worldwide distribution [13–15]. *Schizothoracinae* are economically and commercially essential species living in fast-flowing and snowy rivers and streams including the Neelum and Jhelum Rivers in Azad Jammu and Kashmir [13, 16]. Because of its tender flesh and delicious taste, the Schizothorax fish has become an important economic fish and been strongly targeted and overexploited by commercial fishermen, which have led to the decline of Schizothorax fish [17]. Moreover, in recent years, the *Schizothorax* species have suffered a dramatic decline due to overfishing, polluted water, and destruction of their spawning grounds that resulted in fragmentation of the habitat and impeded the migration of fishes [18, 19].

To overcome the declining fish population, the release of captive breeders into the wild has been helpful for effective conservation strategies to enhance the natural fish populations [20]. It is a widely accepted method to enhance the local populations of some fish species like *Percocypris pingi* and Chinese sturgeon (*Acipenser sinensis*) [21]. Similarly, for threatened Schizothorax species, artificially propagated individuals from hatcheries have also been released into the river to improve populations in the wild. However, in this genus, the discrepancies about morphology-based identification, molecular phylogeny, and evolutionary history were frequently observed [22, 23]. Similarly, the available mitochondrial genome data are insufficient for *Schizothorax* species and especially for *S. niger*.

Therefore, genetic characterization of *S. niger* is an essential step for both fundamental science and its conservation strategy. The main purpose of the current study is to get more information about its genome and locate the taxonomic position of *S. niger* within Schizothoracinae. For this purpose, we characterize the mitochondrial protein-coding genes for *S. niger* and its phylogenetic relationship with other Schizothoracinae.

## 2. Materials and Methods

### 2.1. Sample Collection and DNA Extraction

The specimens of *S. niger* were collected from the Jhelum River (34°19′46.3^″^N 73°30′44.8^″^E) with cast nets. All the fish samples were carefully handled to avoid the damages during studies. The Board of Advanced Studies and Research, University of Azad Jammu and Kashmir, Muzaffarabad, permits to conduct this study in Jhelum and Neelum Rivers of Muzaffarabad city. The collected fishes were anesthetized by immersion in 1% benzocaine in water and euthanized with an overdose of benzocaine. Following the analysis, the voucher specimens were preserved in 70% ethanol and deposited to the Zoological Museum Hall at the University of AJK, Pakistan. By following the classifications of Mirza [24] and Jhingran [25], these specimens were identified. All the collected specimens were obtained in compliance with the animal welfare laws, national policy, and local guidelines in Azad Jammu and Kashmir, Pakistan.

Approximately 0.1 g of tissue was sterilized with ethanol and washed three times with distilled water. The total DNA was isolated by a standard phenol-chloroform extraction method of Sambrook and Russell [26]. The extracted DNA was run on 1% agarose gels and visualized with a UV transilluminator. The results were recorded with a gel documentation system and quantified with a spectrophotometer at the 260/280 nm wavelengths. 16 sets of overlapping primers (Table 1) were used for the amplification of 13 protein-coding genes of the mitochondrial genome of *S. niger* with the use of the Primer-3 program. The mitochondrial DNA was amplified with polymerase chain reactions (PCR), which were performed in 25 *μ*l reaction volumes containing 14 *μ*l DMSO water, 3 *μ*l template DNA, 2.5 *μ*l Taq buffer, 0.5 *μ*l dNTPs, 1 *μ*l of each primer (forward and reverse), 2.5 *μ*l magnesium chloride, and 0.5 *μ*l Taq polymerase. For thorough mixing, the reaction mixture was vortexed and centrifuged for 30 s at 8000 rpm. The thermal cycling profile was 95°C for 3 min; 39 cycles of 95°C for 30 s (denature), 53°C for 30 s (anneal); and 72°C for 1 min (extension) and followed by a final extension at 72°C for 10 min. The PCR products were purified with Exo-SapIT (Affymetrix purification kit) before cycle sequencing.

Bidirectional nucleotide (nt) sequencing was performed on an ABI Prism 3100 Genetic Analyzer (PE Applied Biosystems; Foster City, CA, USA) using gene-specific forward and reverse primers. Sequence editing was performed using the BioEdit program (http://www.mbio.ncsu.edu/BioEdit) to determine nucleotide and amino acid variants. The *S. niger* sequences were aligned by using the ClustalW algorithm of the MegAlign program in the LaserGene software package (DNAStar, Inc., Madison, WI). The sequence analyses were carried out using MEGA 6.06 [27] and DnaSP v5 [28] software. The nucleotide sequences with accession numbers (Table 2) are also available on GenBank. PCGs from 23 Schizothorax species retrieved from GenBank were concatenated and aligned using Sequencher and corrected by eye, yielding a total alignment of 11409-12 nucleotides to determine the sequence divergence among them. The nucleotide and amino acid composition, nucleotide substitutions, codon usage pattern, and relative synonymous codon usage (RSCU) of the 13 PCGs were examined with MEGA 6.0. Nucleotide compositional skew was calculated according to the formula: AT‐skew = (A − T)/(A + T) and GC‐skew = (G − C)/(G + C) [29]. The DAMBE software (v7.2.14) was used to calculate the entropy-based substitution saturation and its critical value [30]. The transitions and transversions against the genetic distance were also calculated through this software.

The Maximum Parsimony (MP) method and BEAST v2.6.2 [31] were used to compute the phylogenetic tree. The BEAST XML input file was generated using BEAUti v2.6.2 (part of the BEAST v2.6.2 package) with strict molecular clock approach and Yule process in tree prior. The MCMC chains were run for 10,000,000 generations, parameters were sampled every 1000 generations, and an initial 10% of the samples were discarded as burn-in. The tree results were summarized using TreeAnnotator v2.6.2 (included in the BEAST package), with the maximum clade credibility tree, posterior probability limit set to 0.5, and summarizing mean node heights. The phylogenetic tree was visualized using FigTree v1.4.4 (http://tree.bio.ed.ac.uk/software/figtree/). The DnaSP 5.0 program was used to calculate the total number of haplotypes, haplotype diversity (Hd), and nucleotide diversity (Pi). Divergence times for all branching points were calculated using the maximum likelihood method by Tamura and Nei [32] model in MEGA 6.06.

## 3. Results and Discussions

### 3.1. Protein-Coding Genes' Features

The boundaries between PCGs of the mtDNA were observed by the alignment of their nucleotide and amino acid sequences and locating their start and stop codons by comparing with cyprinid fishes. The mitochondrial genome of *S. niger* coded 13 PCGs with 11409 bps in length. The lengths of PCGs ranged from 165 (ATPase 8) to 1824 bps (ND5) and encode 3801 amino acids. In these PCGs of the *S. niger* fish, 4 genes overlap on the similar strands, while one shown on the opposite one: ATPase 6+8 and ND4+4L overlap by 7 nucleotides. Similarly, ND5-ND6 overlap by 4 nucleotides, while ATP6 and COIII overlap by 1 nucleotide. Among these, ATPase 6 and 8 genes' overlap is widespread in other vertebrate genomes; however, its size in mammals (40–46 bps) is larger as compared to fish (7–10 bps) [33].

Of the13 PCGs, 12 genes used ATG as start codon, while the COI gene is started with GTG. In particular, in teleosts, the start codon is ATG in all PCGs excluding the COI gene [34, 35]. Similar findings were also observed in *S. niger* PCGs, depicting that COI could be an ancient gene in the evolutionary process of mitochondria. Many studies also reported ATG as an initiation codon of COI gene in many animals, such as *Collichthys niveatus*, *Larimichthys crocea*, *Charybdis feriata*, and *Collichthys lucidus* [36–39]. In the case of termination codon, five genes (ND1, ND4L, ND5, COI, and ATP8) were terminated with TAA, ATP8, and COIII with TA, while the rest of six genes (ND2-ND3, ND4, ATP6, COII, and Cytb) have the incomplete stop codons (T--) (Table 3). The stop codons seem to have an ability to be changed in fish mitogenomes, suggesting that it might undergo a rapid evolutionary process [5, 40]. It is widespread in vertebrate mitochondrial PCGs, and it is suggested that the incomplete termination codons are likely due to posttranscriptional modifications such as in polyadenylation [10, 41]. The overall A+T contents were 54.96% and G+C contents were 45.03% of all 13 protein-coding genes (Table 2). The phenomenon of A+T content higher than G+C content was similar to that of *Boleophthalmus pectinirostris*, *Channel catfish*, *Trachurus trachurus*, *Opsariichthys bidens*, and *Odontobutis potamophila* [34, 42–44], depicting the conserved nature of mitochondrial genomes in the process of evolution.

The nucleotide compositions of the 13 mitochondrial protein-coding genes of the *S. niger* fishes are presented in Table 4. The overall nucleotide composition of 12 concatenated PCGs (H-strand coded) was 26.85% for A, 30.9% for C, 17.95% for G, and 24.35% for T, while the L-strand was as follows: A, 36.6 %; C, 30.5 %; G, 18.4 %; and T, 14.6 % (A>C>G>T). In PCGs, though, the 1^st^ codon positions did not show any deflection of nucleotide composition, as reported in the study of Xenocyprinae [45, 46], albeit the 2^nd^ codon position showed lower G (13.26%) and A (18.95%) contents and higher proportion of T (39.83%) contents. Similarly, the lowest G (10.95%) contents are in the 3^rd^ codon position and higher proportion of A residues (38.77%) in H-strand of *S. niger*, which is in agreement with previous studies [47–50].

Gene-wise codon usage patterns of *S. niger* are depicted in Table 5(a). For amino acids with 4-fold degenerate 3^rd^ position, codons ending with A are higher in *S. niger* as compared to those codons ending with C or T. In 2-fold degenerate codons, the proportion of C is greater compared to that of T. Similarly, G is the least common 3^rd^ position base excluding glycine and arginine (here, G is equal to T and C but quite lower than A). These patterns are generally similar across vertebrate groups [10, 33, 48, 49]. In *S. niger*, the total length of PCGs is 11409 bps, showing the similar length to other Schizothoracinae (Table S2), indicating that the mtDNA is quite conserved in cyprinids.

The most frequently used codon is CUA (5.41%) in 13 PCGs. Furthermore, it was observed that these PCGs coded twenty amino acids in *S. niger*, and the commonly used amino acid is leucine (596); however, the least commonly used amino acid is tryptophan (17). The hydrophobic amino acids (Ala, Ile, Leu, Phe, and Val) were greater compared to polar amino acids (Tyr, Cys, Ser, Asp, and Glu) in 13 protein-coding genes of *S. niger*. The RSCU of the 13 PCGs suggested that the overall 3803 codons were observed in *S. niger* and the most overused codon is GCC (1.54%), while the less frequently used codon is GCG (0.41%) (Table 5(b)).

### 3.2. Combined Analysis

In the present study, 11410 bps of 13 protein-coding genes' sequences were obtained from 26 (3 in-group and 23 retrieved from NCBI) highly specialized Schizothoracinae fishes. Details regarding Schizothorax species retrieved from NCBI are available in Table S1. Sequence alignments of 24 haplotypes were observed in Schizothoracinae, showing that 8095 sites out of 11410 (71%) were conserved while 3315 sites were mutated. Out of the mutated sites, 1975 (60%) were parsimony informative polymorphic sites while 1340 were singleton. Transition mutation was higher compared to transversion. The outnumbered transition mutations follow other reports on mitochondrial DNA in teleost fish [51–54].

The number of haplotypes ranged from one (in all the NCBI sequences) to three (in three studied sequences) within the species. The 0-fold degenerate sites are 7114 (62.34%), while the 4-fold degenerate sites are 1402 (12.28%) out of 11410. The average haplotype diversity (Hd) across all samples was 1.00, while the nucleotide diversity (Pi) was 0.232.

A+T and G+C contents of *S. niger* protein-coding genes were calculated and then compared to other members of Schizothoracinae. The overall base composition was 28.36 % A, 28.64% C, 26.01% T, and 16.98% G, with 54.38% AT, respectively, shown in the similar nucleotide composition with the genus Schizothorax (Table S1). In addition, the proportion of conserved amino acids of 13 PCGs of Schizothorax species was also calculated. Five genes (ND1, COII, COIII, ND4L, and Cytb) were found to have more conserved amino acid sites as compared to others. Among these genes, COI was less conserved as compared to the abovementioned genes; moreover, 73.11% amino acid sites of this were invariable (Table S2).

The interspecific K2P distances ranged from 0.06 to 7.77% (mean 1.105%) for the protein-coding gene. Because of the sequence similarity, the intraspecific distances were 0.00 in studied samples of *S. niger* (SN-01 to SN-03), which could not represent the intraspecific distances of this species, while these sequences show the 0.06% intraspecific K2P distance with *S. niger* (NC-022866.1) retrieved from NCBI. The interspecific divergence of *S. waltoni* to other species is the maximum (5.82-7.77%) as compared to other interspecific differences. The details of species genetic distances are displayed in Table 6.

The best fit model to sequence evolution was selected in GTR+I+G by the Akaike information criteria (AICc) (90532.960). Most of the mutation events were transitions as compared to transversions. The more frequent transitions occur at the GA level (39.19%) while the CT transitions were less frequent (Table S3). Similarly, transition/transversion bias (*R*) is 11.21.

### 3.3. Mitochondrial Genome Evolution

Phylogenetic analysis was used to estimate relationships among *S. niger*, 23 other Schizothorax species, and one outgroup (retrieved from NCBI) to assess historical information content of mitochondrial genomes.

### 3.4. Parsimony Analysis

**Figure 1:**
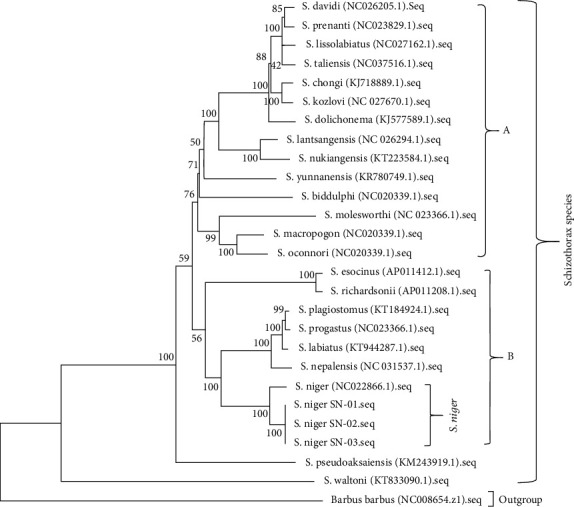
The phylogenetic relationship for fish of the Schizothoracinae with *B. barbus* (outgroup species) using the Maximum Parsimony method.

The evolutionary history was inferred using the Maximum Parsimony method. The most parsimonious tree with length = 7602 is shown. The consistency index is 0.555 (0.447), the retention index is 0.669 (0.669), and the composite index is 0.371 (0.299) for all sites and parsimony informative sites (in parentheses) when *Barbus barbus* was designated as the outgroup (Figure 1). These species created two main clades (A & B) that support maximum bootstrap values. In the tree topology, the monophyly of *S. davidi*+*S. prenanti*, *S. lissolabiatus*+*S. taliensis*, and *S. chongi*+*S. kozlovi* forms a subcluster with *S. dolichonema* with maximum bootstrap support while *S. dolichonema* form the sister group with *S. lantsangensis* and *S. nukiangensis* with 100% bootstrap support. Similarly, *S. molesworthi*+*S. macropogon*+*S. oconnori* form the single subcluster and the base of the group with 99 and 100% bootstrap support. *S. yunnanensis* and *S. biddulphi* do not show a close relationship with other species.

**Figure 2:**
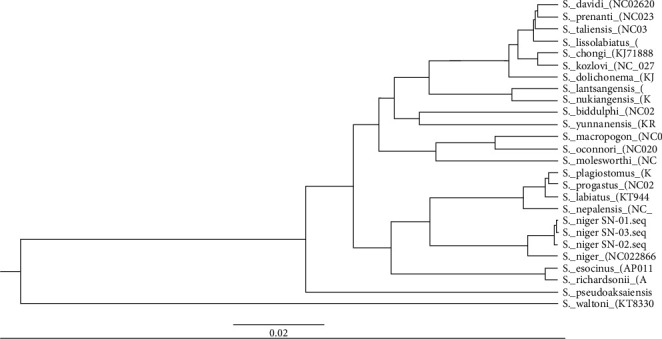
Tree topology is derived from BEAST (FigTree) for the Schizothoracinae.

In clad B, *S. niger* forms a close link with *S. nepalensis*+*S. labiatus*+*S. progastus*+*S. plagiostomus* with maximum (99 and 100%) bootstrap support. These species also show a close relationship with *S. esocinus*+*S. richardsonii*. *S. pseudoaksaiensis* and *S. waltoni* remained separate and do not show the close link with other species. Similarly, *B. barbus* was used as an outgroup and positioned as the base of the tree. Our findings are somewhat similar to the results of Khan et al. [54] and Bibi and Khan [55]. The tree generated in BEAST v2.6.2 through FigTree v1.4.4 (Figure 2) was not identical to the MP in the branching order of Schizothoracinae fishes. As shown in Figure 2, *S. richardsonii* and *S. esocinus* form the separate group from *S. plagiostomus*, *S. progastus*, *S. labiatus*, and *S. nepalensis* and remain at the base *of S. niger*.

**Figure 3:**
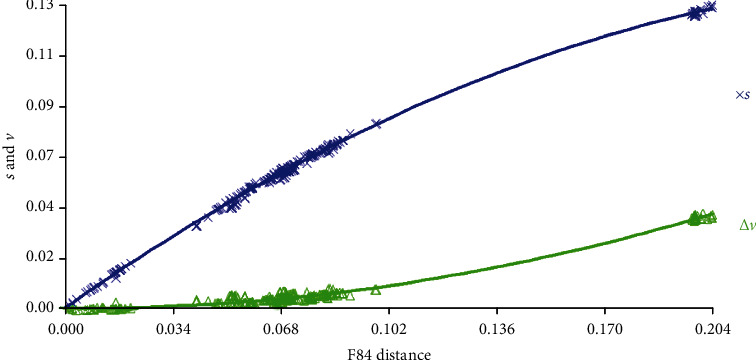
Substitution saturation plot of protein-coding genes of *S. niger*. The number of transitions (*s*) and transversions (*v*) is plotted against F84 genetic distance. A linear correlation is sustained for both transitions and transversions as expected in the absence of saturation.

Using DAMBE, the substitution saturation was assessed for protein-coding genes of *S. niger*. In these genes, no saturation was observed as shown by a linear correlation when the transitions and transversions were plotted against genetic distance (Figure 3). Similarly, the rate of transitional substitutions was higher than that of transversions; similar findings were also reported from Barik et al. [56]. It was also confirmed from a significantly higher (*P* < 0.001) Iss.c value of symmetrical (0.849) and asymmetrical (0.631) as compared to Iss values (0.085). These results depicted that the nucleotide substitutions are not saturated and the data is suitable for phylogenetic study also reported by Li et al. [57] while studying the problematic Cytb gene sequences of fishes from NCBI.

**Figure 4:**
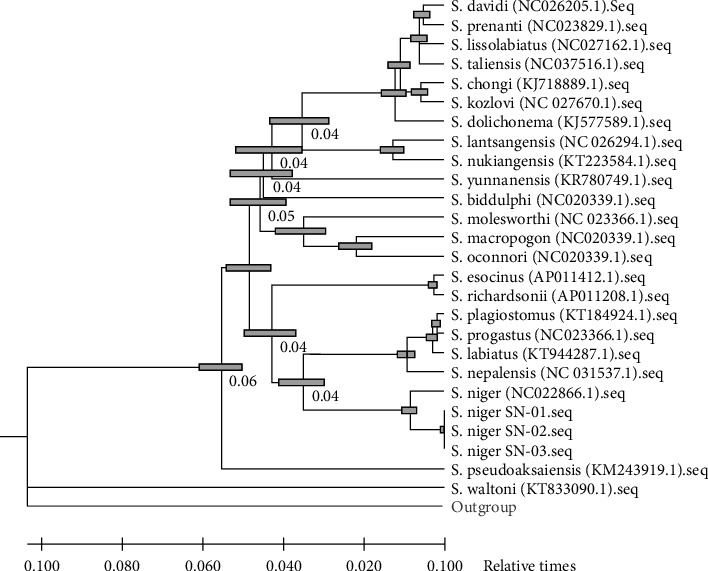
Relative time of divergence was estimated for the Schizothorax species by the maximum likelihood method. Branch lengths are proportional to divergence times (MY).

Through the molecular analysis, the branching time is also estimated along the branching order. The molecular clock estimates of Schizothoracinae fishes are based on a sequence divergence rate of approximately 2.0% per MY [46]. Applying the abovementioned sequence divergence rate, the divergence between *S. niger* and other Schizothorax species (*S. plagiostomus*, *S. progastus*, *S. labiatus*, and *S. nepalensis*) is 0.04%. Similarly, other Schizothoracinae species also show the sequence divergence rate ranging from 0.04 to 0.06 MY as shown in Figure 4. Bars around each node represent 95% confidence intervals which were computed using the method described in Tamura et al. [27]. Our study did not show any conflict with the geological event that causes the uplifting of Himalaya in the late Pliocene to middle Pleistocene (0.5 MY BP) [58].

This is the first study to report genetic data on *S. niger* from the cold water bodies of Azad Jammu and Kashmir, where there is a need to devise conservation and management plans for the exploited cold-water fish species. Through this study, the genetic diversity and phylogenetic relationships among cyprinids and especially of the focal endemic *S. niger* are well explained. Their low intraspecific divergence may cause a threatening condition, and policy actions promoting conservation must be taken immediately. Natural populations need to be maintained at a size sufficient to retain genetic diversity, as this helps to minimize their risk of extinction. Hence, it needs to be spotlighted to conserve the data of genetic diversity. It is mandatory to prevent overfishing, particularly to prohibit fishing throughout the reproductive season. The authors recommend using protein-coding and other mtDNA regions to investigate the genetic divergence and phylogenetic studies of other fish species in freshwater ecosystems in the world.

## Figures and Tables

**Table 1:** Primers used for amplification of the protein-coding genes of *S. niger*.

Primer name	Forward (5′-3′)	Reverse (5′-3′)	*T*_m_
COX1-F1	5′ AACCACAAAGACATTGGTAC 3′	5′ GGTGTCCAAAGAATCAGAAT 3′	54
COX1-F2	5′ ATTCTGATTCTTTGGACACC 3′	5′ TTAATTTGATTGAATTTGAACAA 3′	53
COX11	5′ ACACAACTAGGATTCCAAGAC 3′	5′ AGGCGTCTTCTAGTATTAGTG 3′	60
COX111	5′ ATGGCCCACCAAGCACATGC 3′	5′ TATGAGCCTCATCAATAGATA 3′	60
*Cyt b-F1*	5′ ACCACTATGGCAAGCCTACG 3′	5′ AGGAGATGTAAGATGGTTGCG 3′	60
Cyt b-F2	5′ TGCATTTCACTTCCTACTGCC 3′	5′ GGGCAAGCTCATTCTAGTGC 3′	60
ATPase 6	5′ TTGCGATCGCAATTGCACTC 3′	5′ GTACGGCAGCAGTTAAGATT 3′	59
ATPase 8	5′ GGCCCTTGATTCGCAATTTT 3′	5′ CTATCATGGTCAGTCTCAGG 3′	59
ND1	5′ ATGYTAAACATCYTAATTAC 3′	5′ CAGGGCGGTGTGTCATAGCA 3′	57
ND2	5′ CCATACGTACTTGCAATCCT 3′	5′ AGTAATCCTAGGGTAGACACG 3′	60
ND3	5′ ATGAATCTRATTATAACMAT 3′	5′ ATTCTGCTCATTYTAAGCC 3′	52
ND4	5′ CAACCGCACATAGCCTCCT 3′	5′ GTTGGCTAGGTTGGCGATG 3′	60
ND4L	5′ ATGACACCCGTACAYTTCAG 3′	5′ TTAGCATTGTAGAAGRTTAA 3′	55
ND5-F1	5′ ATGACACTAATTATACACTC 3′	5′ GTGGAGGAATGCTAGTTGTGG 3′	58
ND5-F2	5′ CCACAACTAGCATTCCTCCAC 3′	5′ GGTTAAGTATGTTTTGATTAT 3′	58
ND6	5′ ACGAAGAGTCCCGCGGCTTAA 3′	5′ GACCTATTTCATGTTTCTGTT 3′	61

**Table 2:** Characteristics of studied samples used in this study.

Species	Gene name	NCBI accession no.			A+T	G+C
		*S. niger* SN-01	*S. niger* SN-02	*S. niger* SN-03		
*S. niger*	NDI	MN264181	MN264182	MN264183	50.2	49.8
	ND2	MN264184	MN264185	MN264186	52.2	47.9
	COI	MN264169	MN264170	MN264171	55.5	44.4
	COII	MN264175	MN264176	MN264177	57.6	42.4
	ATP8	MN264205	MN264206	MN264207	63.6	36.3
	ATP6	MN264202	MN264203	MN264204	58.2	41.8
	COIII	MN264178	MN264179	MN264180	54.3	45.8
	ND3	MN264187	MN264188	MN264189	56.5	43.6
	ND4L	MN264193	MN264194	MN264195	52.5	47.4
	ND4	MN264190	MN264191	MN264192	54.6	45.4
	ND5	MN264196	MN264197	MN264198	54.8	45.2
	ND6	MN264172	MN264173	MN264174	51.2	48.9
	CYTB	MN264199	MN264200	MN264201	53.3	46.6

**Table 3:** Characteristics of the protein-coding genes of *S. niger*.

Gene	Size (bps)	Codon			Intergenic nucleotide^a^ (bps)	Strand^b^
		Start	Stop	Amino acid		
NDI	975	ATG	TAA	319	0	H
ND2	1045	ATG	T--	340	0	H
COI	1551	GTG	TAA	500	0	H
COII	691	ATG	T--	225	0	H
ATP8	165	ATG	TA-	49	-7	H
ATP6	684	ATG	TAA	223	-1	H
COIII	785	ATG	TA-	249	0	H
ND3	349	ATG	T--	112	0	H
ND4L	297	ATG	TAA	97	-7	H
ND4	1380	ATG	T--	445	0	H
ND5	1824	ATG	TAA	596	-4	H
ND6	522	ATG	TAA	159	0	L
CYTB	1141	ATG	T--	367	0	H

^a^Negative values indicate the overlapping nucleotides. ^b^H and L indicate that the gene is encoded by that strand.

**Table 4:** The base composition of the protein-coding genes of *S. niger* (the genes which are encoded by the L-strand are converted to complementary strand sequences).

Region	Base composition (%)				1st position				2nd position				3rd position			
	A	T	C	G	A	T	C	G	A	T	C	G	A	T	C	G
H-strand coded																
ND1	24.4	25.8	30.3	19.5	22.8	19	29.8	28	18.2	41	29.2	11.7	32.3	17	31.7	18.8
ND2	29.3	22.9	31.5	16.4	31.8	16	28.4	23.5	15.5	37	35.3	12.1	40.5	15	30.7	13.5
COI	26.5	29.0	26.0	18.4	24.6	22	23.2	30.6	18.4	40	26.3	14.9	36.6	25	28.6	9.9
COII	30.4	27.2	25.8	16.6	23.4	19	25.5	31.6	27.8	38	23.5	10.9	40	24	28.3	7.4
ATP8	33.9	29.7	24.2	12.1	32.7	31	20	16.4	23.6	31	32.7	12.7	45.5	27	20	7.3
ATP6	29.4	28.8	26.6	15.2	29.4	18	28.9	23.2	14.9	47	26.8	11	43.9	21	24.1	11.4
COIII	27.5	26.8	28.2	17.6	20.6	26	26	27.9	21.4	36	26	16.4	40.6	18	32.6	8.4
ND3	28.1	28.4	28.7	14.9	21.4	25	29.1	24.8	17.2	45	25	12.9	45.7	16	31.9	6.9
ND4	28.4	26.2	28.6	16.8	29.1	22	28.9	20.4	16.1	41	27.6	15.7	40	16	29.3	14.3
ND4L	24.9	27.6	30.6	16.8	21.2	25	28.3	25.3	14.1	40	29.3	16.2	39.4	17	34.3	9.1
ND5	30.0	24.8	29.8	15.4	33.4	18	25.3	23.2	20.2	40	27.8	11.5	26.3	16	36.3	11.5
Cytb	25.9	27.4	28.9	17.7	23.4	23	26.2	27	20	42	25	13.2	34.5	17	35.5	12.9
Average	26.85	24.35	30.9	17.95	26.15	22	26.63	25.15	18.95	39.83	27.87	13.26	38.77	19.08	30.27	10.95
L-strand coded																
ND6	36.6	14.6	30.5	18.4	37.9	22	23	17.2	43.7	11	23	22.4	28.2	11	45.4	15.5

**Table:** (a) Gene-wise codon usage patterns in *S. niger*

Amino acid	Codon	ND1	ND2	COI	COII	ATP8	ATP6	COIII	ND3	ND4	ND4L	ND5	ND6	Cytb	Amino acids
Phe	UUU	5	4	17	4	1	7	10	5	8	3	14	1	14	222
	UUC	12	6	23	5	2	5	13	4	10	5	25	3	16	
Leu	UUA	6	8	7	5	5	14	6	6	17	3	13	1	6	596
	UUG	1	2	0	1	0	0	0	0	2	3	2	0	2	
	CUU	8	8	17	7	0	5	3	0	14	2	12	0	7	
	CUC	13	7	10	1	0	10	7	6	15	3	22	2	7	
	CUA	16	21	24	13	0	12	11	11	28	9	38	0	23	
	CUG	16	20	6	1	0	5	4	3	12	2	9	0	16	
Ile	AUU	11	12	27	13	5	14	9	5	17	2	24	1	10	385
	AUC	12	13	12	4	1	9	8	3	20	0	24	2	19	
	AUA	6	11	14	9	0	5	7	3	18	4	19	7	5	
Met	AUG	4	4	10	3	1	5	2	1	9	2	13	1	6	61
Val	GUU	3	1	10	7	0	4	2	2	5	1	7	0	5	217
	GUC	3	4	5	3	0	1	3	1	1	0	8	0	10	
	GUA	6	7	21	10	1	11	6	0	8	1	9	1	11	
	GUG	11	1	6	1	1	1	4	2	3	0	7	0	2	
Ser	UCU	3	3	9	4	0	1	3	1	4	1	2	0	4	238
	UCC	4	6	3	7	2	0	2	3	9	3	9	1	6	
	UCA	8	7	14	2	1	3	5	1	10	2	15	2	8	
	UCG	3	0	1	0	0	1	1	0	1	0	1	0	2	
	AGU	0	0	0	0	1	0	0	0	2	0	1	2	0	
	AGC	4	8	5	3	0	4	3	0	6	3	9	8	1	
Pro	CCU	4	2	5	1	1	2	0	0	2	0	5	0	1	224
	CCC	10	7	9	3	1	3	5	2	7	2	9	11	5	
	CCA	8	8	9	7	6	10	8	6	13	0	13	3	15	
	CCG	2	2	5	2	0	2	0	0	5	0	2	1	0	
Thr	ACU	1	7	6	2	4	3	3	1	3	0	5	2	2	309
	ACC	7	20	8	5	1	2	5	4	16	5	24	7	9	
	ACA	9	18	21	3	0	12	13	4	17	3	23	0	8	
	ACG	1	1	1	2	0	2	1	0	5	0	8	3	2	
Ala	GCU	2	6	7	3	1	2	3	0	2	2	4	1	5	333
	GCC	20	12	22	5	0	9	11	3	17	4	30	6	12	
	GCA	11	19	11	6	1	6	7	4	11	7	15	2	11	
	GCG	2	5	5	2	0	3	1	0	5	0	4	1	5	
Tyr	UAU	8	3	5	5	0	3	5	1	6	0	4	1	3	111
	UAC	4	5	14	4	0	2	7	1	7	0	7	5	11	
Ter	UAA	1	0	1	0	0	1	0	0	0	1	1	10	0	23
	UAG	0	0	0	0	1	0	0	0	0	0	0	7	0	
His	CAU	2	2	5	2	1	1	5	0	6	1	2	5	5	112
	CAC	3	4	14	8	1	2	11	1	5	3	11	5	7	
Gln	CAA	6	13	8	7	1	8	9	3	11	2	19	5	6	109
	CAG	1	1	0	1	0	0	0	0	4	1	1	2	0	
Asn	AAU	5	2	8	4	1	4	0	1	4	0	12	2	4	132
	AAC	7	6	7	2	1	6	1	2	6	2	19	12	14	
Lys	AAA	7	6	8	3	3	1	1	1	8	0	18	8	7	90
	AAG	0	3	0	1	0	0	1	0	3	0	4	5	2	
Asp	GAU	2	0	4	2	0	0	1	2	0	0	3	1	3	77
	GAC	2	4	10	10	1	1	4	2	3	1	10	4	7	
Glu	GAA	7	3	9	14	2	3	7	5	9	2	6	1	7	103
	GAG	4	2	2	1	1	2	3	1	2	1	6	3	0	
Cys	UGU	0	1	0	0	0	0	1	0	0	1	0	0	0	28
	UGC	0	0	1	2	0	0	1	1	5	2	5	5	3	
Ter	UGA	5	8	16	5	5	4	12	4	15	1	11	2	11	99
Trp	UGG	3	3	1	0	0	1	0	1	5	0	1	0	2	17
Arg	CGU	0	0	1	1	0	0	2	0	0	2	0	1	0	84
	CGC	1	1	1	2	0	0	0	2	2	0	1	3	2	
	CGA	4	3	4	3	0	5	3	0	8	1	6	2	4	
	CGG	3	0	2	0	0	1	0	0	1	0	4	0	2	
	AGA	0	0	0	0	0	0	0	0	0	0	0	5	0	
	AGG	0	0	0	0	0	0	0	0	0	0	0	1	0	
Gly	GGU	2	2	8	1	0	1	1	0	2	2	1	2	2	231
	GGC	1	4	4	1	1	1	4	2	6	1	8	5	6	
	GGA	5	9	22	5	0	5	11	5	11	3	15	0	9	
	GGG	10	3	12	2	0	3	5	0	9	0	8	3	8	
Total		325	348	517	230	55	228	261	116	460	99	608	174	380	3801

**Table:** (b) The relative synonymous codon usage of 13 PCGs in the *S. niger*

Amino acid	Codon	Number	Frequency (%)	Amino acid	Codon	Number	Frequency (%)
Phe	UUU	52	0.84	Tyr	UAU	104	1.09
Phe	UUC	72	1.16	Tyr	UAC	86	0.91
Leu	UUA	97	1.47	∗	UAA	98	1.21
Leu	UUG	62	0.94	∗	UAG	85	1.05
Leu	CUU	59	0.89	His	CAU	74	0.91
Leu	CUC	42	0.63	His	CAC	88	1.09
Leu	CUA	72	1.09	Gln	CAA	110	1.31
Leu	CUG	65	0.98	Gln	CAG	58	0.69
Ile	AUU	72	1.09	Asn	AAU	90	1.09
Ile	AUC	62	0.94	Asn	AAC	75	0.91
Ile	AUA	64	0.97	Lys	AAA	83	1.36
Met	AUG	34	1	Lys	AAG	39	0.64
Val	GUU	11	0.57	Asp	GAU	50	1.06
Val	GUC	22	1.14	Asp	GAC	44	0.94
Val	GUA	21	1.09	Glu	GAA	46	1.02
Val	GUG	23	1.19	Glu	GAG	44	0.98
Ser	UCU	84	1.22	Cys	UGU	39	0.94
Ser	UCC	98	1.42	Cys	UGC	44	1.06
Ser	UCA	99	1.44	∗	UGA	60	0.74
Ser	UCG	48	0.7	Trp	UGG	66	1
Pro	CCU	102	1.11	Arg	CGU	26	0.79
Pro	CCC	114	1.25	Arg	CGC	26	0.79
Pro	CCA	94	1.03	Arg	CGA	27	0.82
Pro	CCG	56	0.61	Arg	CGG	40	1.22
Thr	ACU	77	1.01	Ser	AGU	35	0.51
Thr	ACC	104	1.37	Ser	AGC	49	0.71
Thr	ACA	83	1.09	Arg	AGA	41	1.25
Thr	ACG	40	0.53	Arg	AGG	37	1.13
Ala	GCU	45	0.96	Gly	GGU	24	0.78
Ala	GCC	72	1.54	Gly	GGC	31	1.01
Ala	GCA	51	1.09	Gly	GGA	31	1.01
Ala	GCG	19	0.41	Gly	GGG	37	1.2

^∗^Represents termination codon.

**Table 6:** Interspecific K2P pairwise distance (%) based on protein-coding gene.

*Sr*.	Species name	1	2	3	4	5	6	7	8	9	10	11	12	13	14	15	16	17	18	19	20	21	22	23	24	25	26
1	*S. biddulphi*																										
2	*S. chongi*	0.88																									
3	*S. davidi*	1.04	0.01																								
4	*S. dolichonema*	1.19	0.04	0.02																							
5	*S. esocinus*	0.96	3.49	3.80	3.94																						
6	*S. kozlovi*	1.01	0.01	0.00	0.01	3.68																					
7	*S. labiatus*	0.44	0.08	0.13	0.21	2.62	0.13																				
8	*S. lantsangensis*	1.23	0.06	0.04	0.00	3.96	0.02	0.24																			
9	*S. lissolabiatus*	0.95	0.01	0.01	0.05	3.69	0.02	0.10	0.08																		
10	*S. macropogon*	0.42	0.09	0.14	0.21	2.51	0.13	0.01	0.24	0.12																	
11	*S. molesworthi*	0.01	0.76	0.92	1.06	1.11	0.89	0.36	1.11	0.83	0.34																
12	*S. nepalensis*	0.23	0.25	0.34	0.46	2.08	0.34	0.05	0.50	0.27	0.06	0.16															
13	*S. niger*	0.53	0.12	0.18	0.30	2.89	0.20	0.04	0.36	0.11	0.07	0.43	0.07														
14	*S. niger SN-01*	0.74	0.01	0.04	0.10	3.27	0.04	0.04	0.13	0.01	0.05	0.63	0.17	0.06													
15	*S. niger SN-02*	0.74	0.01	0.04	0.10	3.27	0.04	0.04	0.13	0.01	0.05	0.63	0.17	0.06	0.00												
16	*S. niger SN-03*	0.74	0.01	0.04	0.10	3.27	0.04	0.04	0.13	0.01	0.05	0.63	0.17	0.06	0.00	0.00											
17	*S. nukiangensis*	0.92	0.03	0.04	0.02	3.39	0.02	0.13	0.02	0.07	0.12	0.82	0.33	0.25	0.08	0.08	0.08										
18	*S. oconnori*	0.21	0.23	0.32	0.41	1.98	0.30	0.04	0.45	0.27	0.04	0.16	0.02	0.11	0.16	0.16	0.16	0.27									
19	*S. plagiostomus*	0.49	0.06	0.11	0.16	2.67	0.10	0.00	0.19	0.08	0.01	0.40	0.07	0.06	0.03	0.03	0.03	0.09	0.06								
20	*S. prenanti*	0.85	0.00	0.01	0.05	3.44	0.01	0.07	0.07	0.01	0.08	0.73	0.23	0.11	0.01	0.01	0.01	0.04	0.22	0.05							
21	*S. progastus*	0.47	0.07	0.12	0.19	2.66	0.11	0.00	0.22	0.09	0.00	0.38	0.06	0.05	0.03	0.03	0.03	0.11	0.05	0.00	0.06						
22	*S. pseudoaksaiensis*	0.39	0.11	0.17	0.25	2.50	0.16	0.01	0.29	0.13	0.01	0.33	0.05	0.05	0.06	0.06	0.06	0.16	0.03	0.02	0.09	0.01					
23	*S. richardsonii*	1.19	3.87	4.19	4.31	0.02	4.05	2.96	4.33	4.09	2.84	1.36	2.41	3.28	3.65	3.65	3.65	3.74	2.28	3.02	3.82	3.00	2.83				
24	*S. taliensis*	0.97	0.00	0.00	0.02	3.62	0.00	0.11	0.03	0.01	0.12	0.85	0.31	0.18	0.03	0.03	0.03	0.02	0.28	0.09	0.01	0.10	0.14	4.00			
25	*S. waltoni*	6.69	6.71	6.74	6.22	6.34	6.46	6.77	5.97	7.08	6.49	6.70	6.97	7.77	7.06	7.06	7.06	5.82	6.51	6.49	6.77	6.67	6.85	6.21	6.55		
26	*S. yunnanensis*	0.57	0.07	0.11	0.21	2.97	0.12	0.02	0.25	0.07	0.04	0.47	0.08	0.01	0.02	0.02	0.02	0.17	0.10	0.03	0.06	0.02	0.04	3.35	0.11	7.37	

## Data Availability

The data that support the findings of this study are openly available in the repository under Accession: MN264169-MN264207 (https://www.ncbi.nlm.nih.gov/nuccore/MN264169-MN264207).

## Supplementary Materials

Supplementary Materials Table S1: composition and skewness in the PCGs of Schizothorax mitogenomes. Table S2: proportion of conserved amino acid sites in 13 PCGs of Schizothorax species. Table S3: maximum likelihood estimate of substitution matrix in Schizothoracinae. Rates of different transitional substitutions are shown in bold, and those of transversion substitutions are shown in *italics*.
