# Supplementary material for: Sequencing and Characterization of Mitochondrial Protein-Coding Genes for Schizothorax niger (Cypriniformes: Cyprinidae) with Phylogenetic Consideration
Source: Biomed Res Int. 2020 Dec 7;2020:5980135. doi: 10.1155/2020/5980135 (PMC7787735; doi:10.1155/2020/5980135)
Supplement: Supplementary Materials — Table S1: composition and skewness in the PCGs of Schizothorax mitogenomes. Table S2: proportion of conserved amino acid sites in 13 PCGs of Schizothorax species. Table S3: maximum likelihood estimate of substitution matrix in Schizothoracinae. Rates of different transitional substitutions are shown in bold, and those of transversion substitutions are shown in italics. [file 5980135.f1.docx]

Table S1. Composition and skewness in the PCGs of Schizothorax mitogenomes

| Species | A | C | G | T | A+T % | AT skew | GC skew | Size | Genbank | References |
| --- | --- | --- | --- | --- | --- | --- | --- | --- | --- | --- |
| *S. niger-*01 | 28.36 | 28.64 | 16.98 | 26.01 | 54.38 | 0.043 | -0.256 | 11409 | Mention in Table 2 | Present study |
| *S. niger-*02 | 28.36 | 28.64 | 16.98 | 26.01 | 54.38 | 0.043 | -0.256 | 11409 | Mention in Table 2 | Present study |
| *S. niger-*03 | 28.36 | 28.64 | 16.98 | 26.01 | 54.38 | 0.043 | -0.256 | 11409 | Mention in Table 2 | Present study |
| *S. biddulphi* | 29.01 | 28.16 | 16.34 | 26.49 | 55.50 | 0.045 | -0.266 | 11409 | NC017873.1 | Gong et al., 2012 |
| *S. chongi* | 28.25 | 28.62 | 17.07 | 26.06 | 54.30 | 0.040 | -0.253 | 11410 | KJ718889.1 | Que et al., 2014 |
| *S. davidi* | 28.17 | 28.66 | 17.14 | 26.03 | 54.20 | 0.039 | -0.252 | 11410 | NC026205.1 | Wang et al., 2016 |
| *S. dolichonema* | 28.05 | 28.59 | 17.24 | 26.10 | 54.15 | 0.036 | -0.248 | 11412 | KJ577589.1 | Unpublished |
| *S. esocinus* | 29.49 | 27.43 | 15.79 | 27.28 | 56.77 | 0.039 | -0.269 | 11409 | AP011412.1 | Unpublished |
| *S. kozlovi* | 28.16 | 28.61 | 17.15 | 26.08 | 54.24 | 0.038 | -0.250 | 11410 | NC_027670.1 | Unpublished |
| *S. labiatus* | 28.49 | 28.54 | 16.83 | 26.14 | 54.64 | 0.043 | -0.258 | 11410 | KT944287.1 | Unpublished |
| *S. lantsangensis* | 28.00 | 28.58 | 17.26 | 26.16 | 54.16 | 0.034 | -0.247 | 11410 | NC_026294.1 | Unpublished |
| *S. lissolabiatus* | 28.25 | 28.70 | 17.06 | 25.99 | 54.23 | 0.042 | -0.254 | 11410 | NC027162.1 | Yue et al., 2015 |
| *S. macropogon* | 28.49 | 28.46 | 16.86 | 26.19 | 54.68 | 0.042 | -0.256 | 11410 | NC020339.1 | Zhu et al., 2013 |
| *S. molesworthi* | 28.93 | 28.26 | 16.34 | 26.47 | 55.40 | 0.044 | -0.267 | 11410 | MG171194.1 | Zhang, 2019 |
| *S. nepalensis* | 28.65 | 28.53 | 16.59 | 26.22 | 54.88 | 0.044 | -0.265 | 11409 | NC_031537.1 | Unpublished |
| *S. niger* | 28.58 | 28.69 | 16.77 | 25.97 | 54.55 | 0.048 | -0.262 | 11410 | NC022866.1 | Unpublished |
| *S. nukiangensis* | 28.13 | 28.50 | 17.14 | 26.22 | 54.36 | 0.035 | -0.249 | 11410 | KT223584.1 | Li et al., 2015 |
| *S. oconnori* | 28.63 | 28.40 | 16.70 | 26.28 | 54.91 | 0.043 | -0.259 | 11410 | NC_020781.1 | Chen et al., 2014 |
| *S. plagiostomus* | 28.41 | 28.53 | 16.88 | 26.19 | 54.60 | 0.041 | -0.257 | 11411 | KT184924.1 | Unpublished |
| *S. prenanti* | 28.27 | 28.62 | 17.06 | 26.05 | 54.32 | 0.041 | -0.253 | 11410 | NC023829.1 | Chen et al., 2014 |
| *S. progastus* | 28.47 | 28.53 | 16.85 | 26.15 | 54.62 | 0.042 | -0.257 | 11410 | NC023366.1 | Unpublished |
| *S. pseudoaksaiensis* | 28.57 | 28.45 | 16.85 | 26.11 | 54.68 | 0.045 | -0.256 | 11412 | KM243919.1 | Luan et al., 2014 |
| *S. richardsonii* | 29.52 | 27.31 | 15.79 | 27.38 | 56.90 | 0.038 | -0.267 | 11409 | AP011208.1 | Unpublished |
| *S. taliensis* | 28.19 | 28.60 | 17.14 | 26.06 | 54.26 | 0.039 | -0.251 | 11410 | NC037516.1 | Unpublished |
| *S. waltoni* | 26.92 | 27.06 | 17.22 | 28.80 | 55.72 | -0.034 | -0.222 | 11410 | KT833090.1 | Chen et al., 2016 |
| *S. yunnanensis* | 28.48 | 28.67 | 16.83 | 26.02 | 54.50 | 0.045 | -0.260 | 11410 | KR780749.1 | Yue, 2015 |

Table S2. Proportion of conserved amino acid sites in 13 PCGs of Schizothorax species.

| Genes | **No. of nucleotides** | **Number of invariable nucleotide sites** | **No. of amino acids** | **Number of invariable amino acid sites** | **Proportion of invariable amino acid sites (%)** |
| --- | --- | --- | --- | --- | --- |
| ND1 | 975 | 676 | 325 | 287 | 88.31 |
| ND2 | 1046 | 666 | 349 | 219 | 62.75 |
| COI | 1551 | 1215 | 517 | 378 | 73.11 |
| COII | 691 | 554 | 230 | 206 | 89.57 |
| ATP8 | 165 | 135 | 54 | 38 | 70.37 |
| ATP6 | 684 | 475 | 277 | 189 | 68.23 |
| COIII | 785 | 624 | 262 | 235 | 89.69 |
| ND3 | 349 | 245 | 117 | 92 | 78.63 |
| ND4L | 297 | 211 | 99 | 89 | 89.90 |
| ND4 | 1380 | 932 | 460 | 374 | 81.30 |
| ND5 | 1824 | 1252 | 608 | 500 | 82.24 |
| ND6 | 522 | 317 | 174 | 44 | 25.29 |
| Cytb | 1141 | 802 | 380 | 336 | 88.42 |

**Table S3.** Maximum Likelihood Estimate of Substitution Matrix in Schizothoracinae. Rates of different transitional substitutions are shown in **bold** and those of transversions substitutions are shown in *italics*.

|  | **A** | **T/U** | **C** | **G** |
| --- | --- | --- | --- | --- |
| **A** | - | *0.80* | *0.86* | **23.20** |
| **T/U** | *0.86* | - | **16.39** | *0.51* |
| **C** | *0.86* | **15.20** | - | *0.51* |
| **G** | **39.13** | *0.80* | *0.86* | - |
